# Adaptations of an online cognitive-behavioral therapy intervention for binge type eating disorders in publicly-insured and uninsured adults: a pilot study

**DOI:** 10.1186/s12889-025-22494-w

**Published:** 2025-04-07

**Authors:** Siena S. Vendlinski, Agatha A. Laboe, Peyton Crest, Claire G. McGinnis, Molly F. Steinhoff, Denise E. Wilfley, C. Barr Taylor, Ellen E. Fitzsimmons-Craft, Erin C. Accurso

**Affiliations:** 1 Department of Pediatrics, University of California, San Francisco, San Francisco, CA USA; 2 Department of Psychology, University of Wisconsin-Madison, Madison, WI USA; 3 Department of Psychology, Rhodes College, Memphis, TN USA; 4 Department of Psychiatry, Washington University School of Medicine, St. Louis, MO USA; 5 Department of Psychiatry and Behavioral Sciences, Stanford University School of Medicine, Stanford, CA USA; 6 Department of Psychological and Brain Sciences, Washington University in St. Louis, St. Louis, USA; 7 Department of Psychiatry and Behavioral Sciences, University of California, San Francisco, San Francisco, CA USA; 8 Philip R. Lee Institute for Health Policy Studies, University of California, San Francisco, San Francisco, USA

**Keywords:** Bulimia nervosa, Binge eating disorder, Eating disorders, Digital intervention, Public insurance, Cognitive behavioral therapy, mHealth

## Abstract

**Background:**

Publicly-insured and uninsured individuals—many of whom are marginalized because of race/ethnicity, ability status, and/or other social identities—experience barriers to accessing evidence-based interventions (EBIs) for eating disorders (EDs). Additionally, EBIs have not been developed with or for diverse populations, exacerbating poor treatment uptake. Mobile technology is well-positioned to bridge this gap and increase access to low-cost, culturally-sensitive EBIs.

**Methods:**

This study leverages a user-centered design approach to adapt an existing coached cognitive-behavioral therapy-based digital program and evaluate its usability in a sample of 11 participants with (sub)clinical binge type EDs who are publicly-insured (*n* = 10) or uninsured (*n* = 1). Participants were primarily non-Latinx White women (*n* = 8). Two semi-structured interviews occurred with participants: one to assess treatment needs and the other to obtain program-specific feedback. Interviews were coded using inductive thematic analysis.

**Results:**

Interview 1 feedback converged on three themes: Recovery Journey, Treatment Experiences, and Engagement with and Expectations for Online Programs. Participants endorsed facing barriers to healthcare, such as poor insurance coverage and a lack of trained providers, and interest in a coach to increase treatment accountability. Interview 2 feedback converged on three themes: Content Development, Participant Experiences with Mental Health, and Real-World Use. Participants liked the content but emphasized the need to improve diverse representation (e.g., gender, body size).

**Conclusions:**

Overall, user feedback is critical to informing adaptations to the original EBI so that the intervention can be appropriately tailored to the needs of this underserved population, which ultimately has high potential to address critical barriers to ED treatment.

**Trial registration:**

This study was reviewed and approved by the Institutional Review Board (IRB) at the University California, San Francisco (IRB #22-35936) and the IRB at Washington University in St. Louis (IRB ID 202304167).

**Supplementary Information:**

The online version contains supplementary material available at 10.1186/s12889-025-22494-w.

## Background

Eating disorders (EDs) are severe psychiatric illnesses characterized by disrupted eating behaviors and excessive preoccupation with weight, food, and body image that interfere with functioning. Binge type EDs (e.g., binge eating disorder, bulimia nervosa, anorexia nervosa binge-purge) are characterized by episodes of subjective or objective overeating with a sense of loss of control (i.e., binge eating). Specifically, binge-purge type EDs feature engagement in compensatory behaviors (e.g., purging, overexercising) following such episodes of binge eating. Binge type EDs affect individuals across race, ethnicity, body size, and socioeconomic status (SES), with bulimia nervosa being more prevalent among Latinx and Black compared to White individuals [1–3]. Unfortunately, < 20% of individuals with EDs ever receive care, with treatment uptake being lowest amongst those who do not identify as female, White, non-Latinx, or high income [4–9].

Low treatment uptake is exacerbated by poor treatment access, particularly for individuals from under-resourced communities, including those with low SES, racial and ethnic minorities, and those with Medicaid insurance (public insurance in the U.S.) [5, 10–13]. Numerous barriers to care are salient, such as the high costs of participating in specialized mental health services [6, 14]. Individuals with public insurance in the U.S. are often unable to access high-quality treatment because providers in publicly-funded settings are not trained in treating EDs [15]. As such, innovative solutions, such as digital interventions, are needed to increase treatment of EDs among individuals who are high risk for health inequities [16].

Digital interventions may be especially beneficial for individuals from lower SES backgrounds given that they reduce logistic and financial barriers to care (e.g., travel, work schedule, flexibility with timing of use). Publicly-insured adults in the U.S. have relatively high digital access, with 86% owning a smartphone and 79% having access to broadband internet in their household [17, 18]. However, standard ED treatments have not yet been adequately tailored to meet the unique needs of individuals from lower SES and racially or ethnically diverse backgrounds.

Cultural adaptation is critical in improving treatment relevance, acceptability, effectiveness, and sustainability of evidence-based interventions (EBIs) [15, 19–21]. Cultural adaptations may be particularly impactful when informed by user-designed approaches (otherwise known as human-centered design or design thinking). User-centered design focuses the design of technology on individuals who will be using it and the contexts in which it will be implemented. It is particularly effective when conducted in iterative phases and generally includes usability testing, which assesses whether the product functions as intended [22]. In line with a user-centered design approach, this study developed and evaluated the usability of a guided self-help, cognitive-behavioral therapy (CBT)-based digital program—a guideline-based, first-line treatment for binge type EDs in adults—for use among individuals with EDs who are publicly-insured or uninsured [23].

## Methods

### Participants and recruitment

Eligibility criteria required that participants were at least 18 years old, screened positive for a clinical or subclinical binge-type ED on the Stanford-Washington University Eating Disorder Screen (SWED), had a BMI ≥ 18.5, and were publicly-insured (e.g., Medicaid, Medi-Cal, or Medicare) or uninsured [24]. Participants were also required to speak and read English, reside in the United States, and have access to an electronic device with Internet access (e.g., smartphone, computer, or tablet). Participants were primarily recruited through the online ED screening tool hosted by the National Eating Disorders Association. Additional recruitment was facilitated through social media, community health partners, and ED advocacy groups. These participants also completed the SWED to determine eligibility. Eligible participants were approached via email and/or telephone as soon as they completed the SWED and were enrolled upon providing informed consent.

### Procedure

Verbal consent was obtained from eligible participants prior to enrollment. Participants completed two semi-structured virtual interviews—a one-hour needs assessment interview and a one-hour usability testing interview (see Supplemental Information for interview guides). Interviews were conducted by a senior research coordinator (SSV) or co-principal investigator (ECA), who both identify as cisgender females. Prior to engaging in the interviews, participants had limited interactions with the interviewers. The senior research coordinator obtained informed consent over the phone, followed by an email exchange to schedule the interview. All interviews were conducted in a private room via videoconferencing with no additional participants present. Interview guides were developed by the co-principal investigators (EFC and ECA), who respectively have expertise in developing digital interventions for EDs and publicly-insured populations with EDs. The interviews focused on potential adaptations to adequately address context and related challenges specific to individuals with EDs with fewer resources (e.g., finances, time, education) and diverse social identities (e.g., gender, race/ethnicity, ability status, and weight status). The methods, results, and discussion in this manuscript are reported in accordance with the COnsolidated criteria for REporting Qualitative research (CORE-Q) Checklist [25].

#### Needs assessment

Needs assessment questions gathered data on past ED treatment experiences (or lack thereof), current goals related to ED recovery, the role of technology in everyday life, and how technology can be harnessed to support mental health generally and ED recovery specifically.

#### Usability testing

Participants who completed the needs assessment interview completed usability testing one to three months later. Usability testing interviews were initially planned as small focus groups, both for efficiency and effectiveness in eliciting a diversity of perspectives and collaborative reflections. One usability testing interview was done in a group of two participants. However, scheduling focus groups across multiple time zones with participants’ limited availability was ultimately not feasible, and the study team had concerns about participants’ capacity to be forthcoming when interviewed about a sensitive topic in group format. Therefore, the remainder of interviews were completed individually. Participants were shown screenshots of different modules and provided feedback on content, intervention design, aesthetics/layout, clarity, accessibility, and perceived usefulness of the intervention.

### Online intervention design

**Fig. 1:**
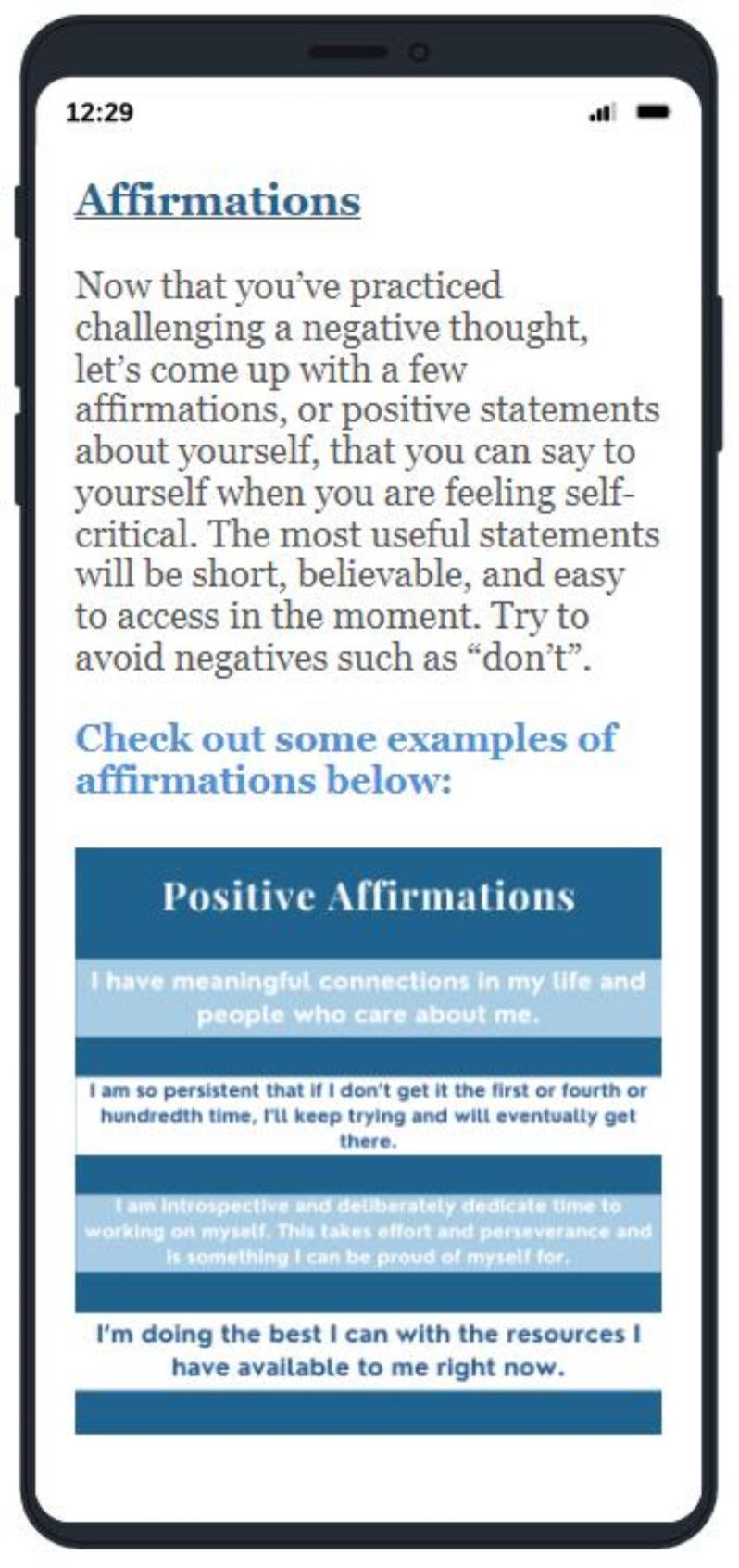
Cognitive-behavioral therapy-informed educational content on cognitive restructuring

**Fig. 2:**
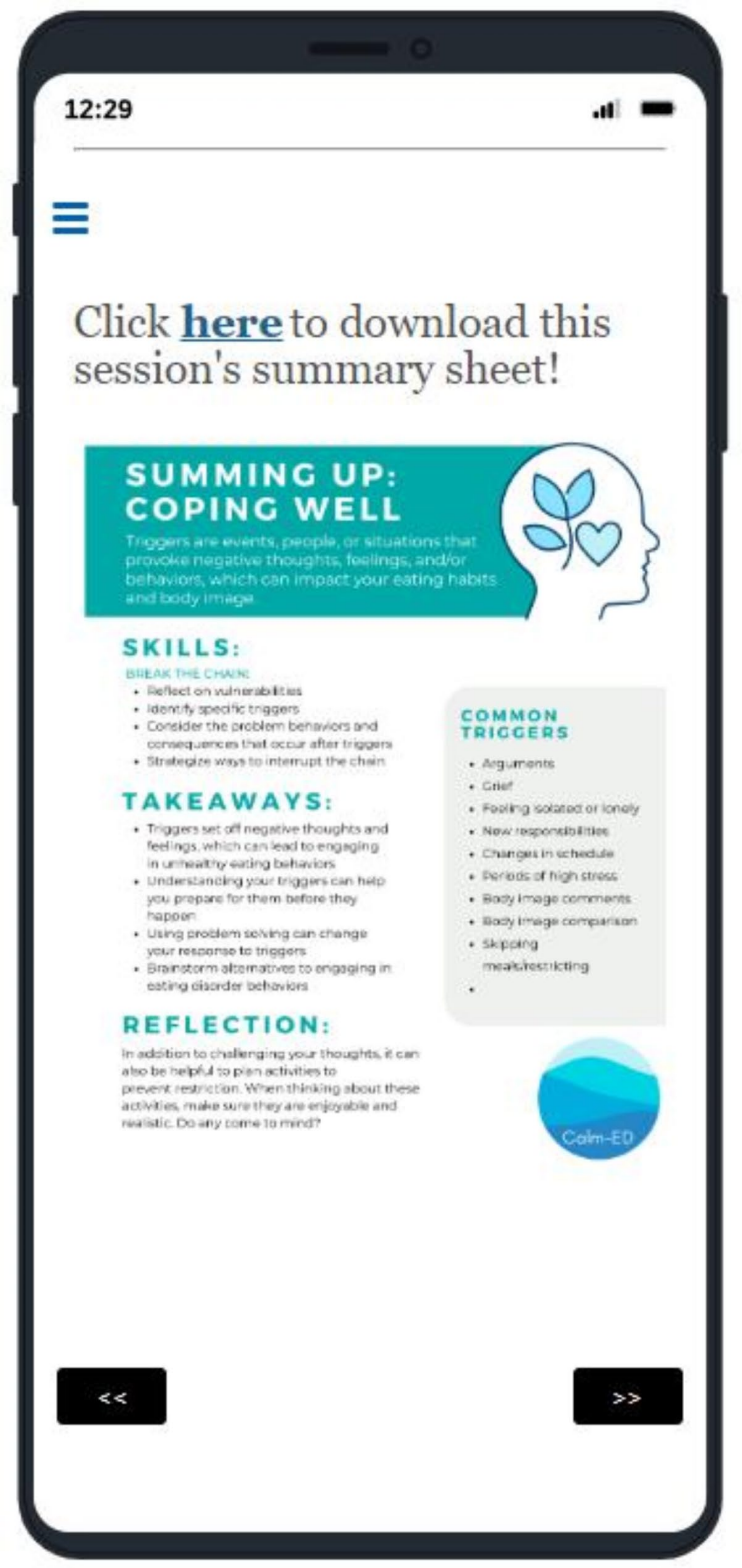
Downloadable PDF summary of session content

**Fig. 3:**
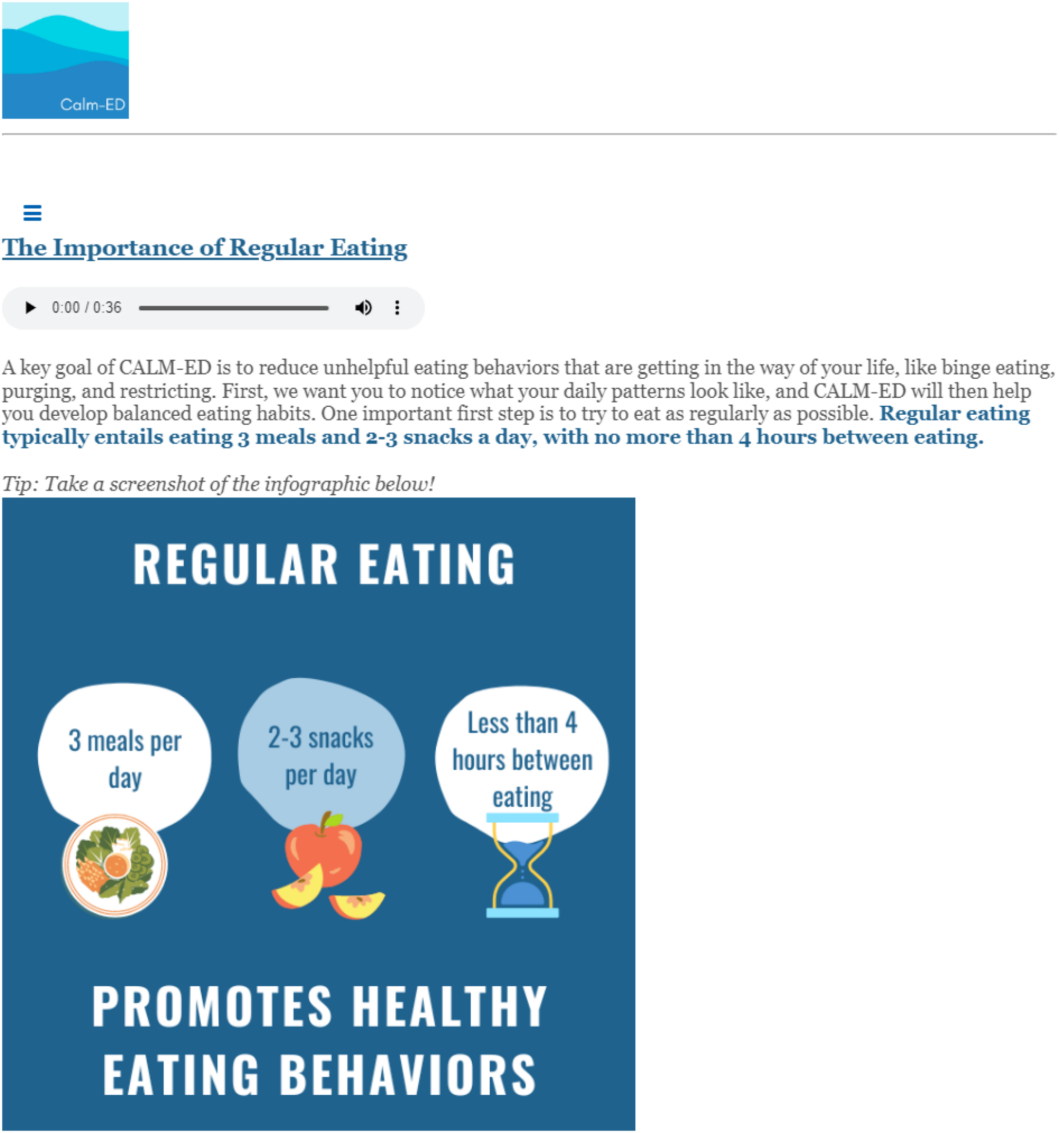
Cognitive-behavioral therapy-informed educational content on the tenets of regular eating

**Fig. 4:**
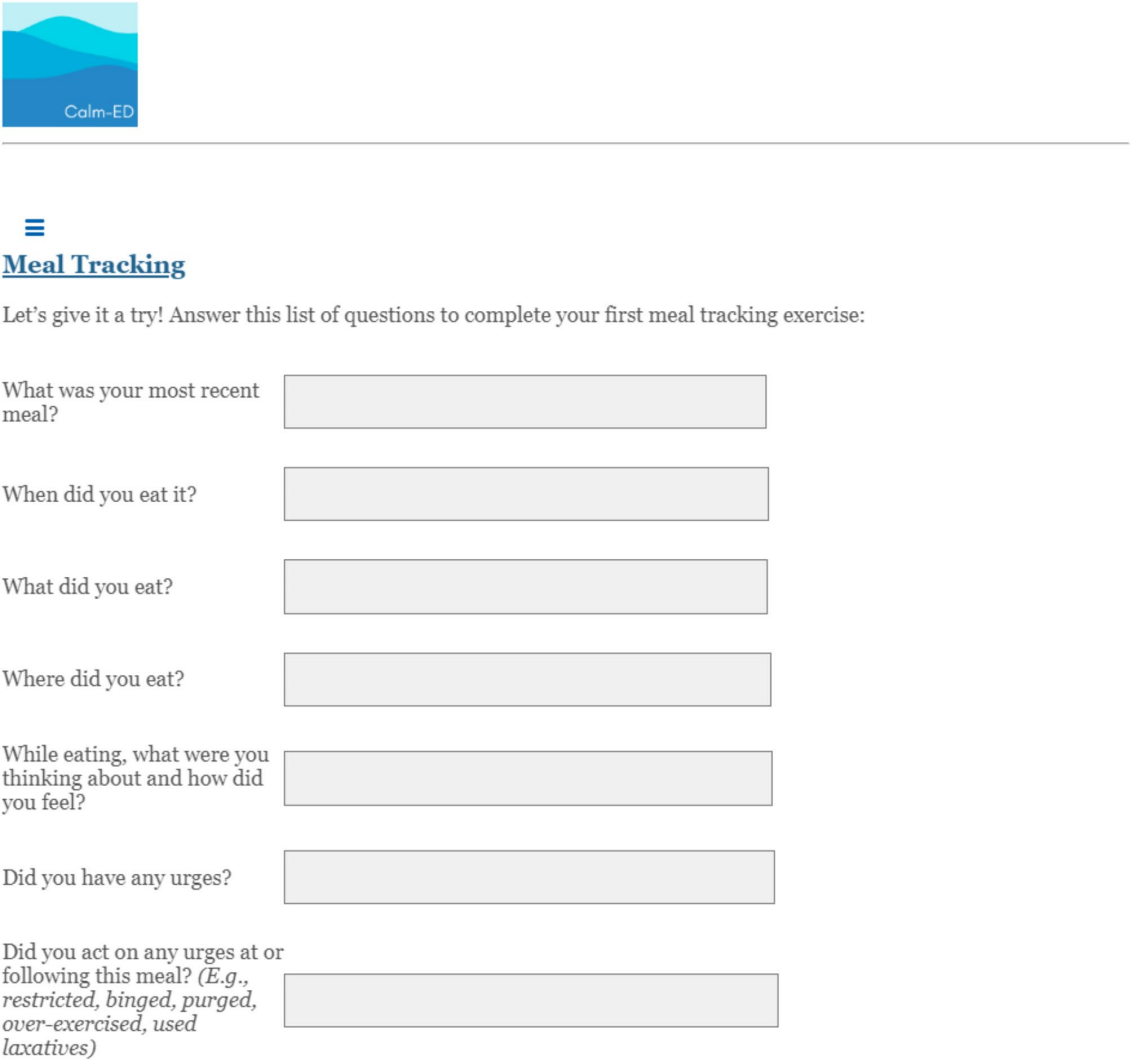
A self-monitoring tool to track food, emotions, urges, and behaviors after meals

The web-based intervention, *Changing Attitudes*,* baLance*,* and Mindfulness for Eating Disorders (CALM-ED)*, was adapted based on an existing CBT-based online program shown to be effective in reducing ED behaviors in a population of college women with binge-type EDs [26–28]. CALM-ED comprises eight CBT-informed modules: Eating Well, Coping Well, Thinking Well, Mind and Body Wellness, Media Wellness, Relationship Wellness, Emotional Wellness, Future Wellness. Each module delivers psychoeducational content with infographics, interactive activities, and supplemental materials (e.g., downloadable PDFs). See Supplemental Information for an overview of each module. Additionally, every time participants log into the program, they are asked to “check-in” about how often they have used skills (e.g., challenging negative thoughts, responding to triggers by engaging in helpful behaviors instead of acting on ED urges) from the most recent module they completed, and are able to describe challenges that may have interfered with utilizing the skills. Hosted on a web-based platform, CALM-ED can be accessed from both mobile devices (e.g., smartphone, tablet) and computers (refer to Figs. 1, 2, 3 and 4 for screenshots).

Eating Well (Session 1) contains crucial, foundational information about the tenets of regular eating (e.g., eating three meals and two to three snacks per day), nutrition, meal planning and tracking, and how to implement regular eating in the context of food insecurity. Though the program does not create personalized meal plans for each participant, it does address the importance of incorporating a mix of proteins, fats, and carbs to feel satiated and meet participants’ nutritional needs. There is also information about the impact of eating low-calorie and low-fat foods that often lack fiber and contain excess sugar, which can increase the likelihood of having a binge episode later in the day. Following this nutritional information, the meal planning tool is presented, which allows participants to plan when, what, and where they will eat at least a day in advance. The meal tracking tool, which is used after meals and snacks, repeats these questions (i.e., when, what, and where they ate) and additionally asks about emotions, urges, and/or behaviors that occurred during or after mealtime. Meal planning and tracking are critical to identifying patterns that may be contributing to binge and purge cycles; for example, planning to eat breakfast at work but not allotting enough time to finish the meal, and then eating more than planned at the next snack. Consistently documenting meals and snacks facilitates awareness of areas to make changes (e.g., planning to eat before going to work) and prevent a binge. Furthermore, because this population is at high risk of experiencing food insecurity, the study team added a section to Session 1 that identifies resources (e.g., Feeding America, Supplemental Nutrition Assistance Program) to obtain adequate nutrition. Examples of meals and snacks provided in the program intentionally centered around affordable and accessible options to make regular eating feasible and sustainable; for example, using frozen or canned vegetables, fruits, and proteins.

### Qualitative thematic analysis

All interviews were recorded and transcribed in real time via Zoom; the interviewer did not take notes during the interviews in order to facilitate full engagement with participants. A member of the study team subsequently reviewed each transcript while listening to the recording to ensure accuracy.

In line with best practices, coders discussed their positionality in respect to the study participants prior to initiating the coding process. The majority of participants were cisfemale adults identifying as White, non-Latinx, and LGBQ+. Most also endorsed living with a chronic health condition, having public insurance or no insurance, and living in a larger body. Based on these characteristics, the coders had both insider and outsider status. Coders had similar characteristics with the sample in their ethnicity, race, gender, and sexual orientation (all coders were non-Latinx White cisgender heterosexual females) and different characteristics with respect to body size, insurance, and health (none of the coders identified as larger-bodied, publicly-insured or uninsured, or were living with a chronic health condition). Finally, the coders’ training background was somewhat homogenous, with 3/4 coders working as post-baccalaureate research coordinators in academic medical centers and 1/4 coders being an undergraduate research assistant.

Then, following steps identified by Braun & Clark, the study team analyzed transcripts through a multi-step process: the study team read the transcripts, created separate codebooks for the needs assessment and usability testing interviews, and coded the transcripts (two independent coders on each transcript) [23, 24]. Coding discrepancies were then identified and resolved by one coder, and these decisions were reviewed by two independent coders to ensure team consensus. The software Dedoose (*Dedoose version 9.0*, 2023) was used to code transcripts.

After the final codes were applied, theme development began [24]. First, codes were grouped into subthemes based on their similarities in the context of the transcripts. Subthemes were then grouped into themes, and the transcripts were reviewed again with these themes in mind. After making the final adaptations, coders named and defined the themes and subthemes.

## Results

### Participants

We expected that the 11 participants enrolled sufficed for the purposes of qualitative analyses since sample sizes above nine often achieve coding saturation [29, 30]. Indeed, coding saturation was achieved in the present study. Adult participants were primarily cisgender females (*n =* 8, 72%) who identified as White and non-Latinx (*n =* 8, 72%) with a household income of less than $40,120 (*n =* 9, 81.8%)—the poverty line for a household of 7 people when these data were collected. One participant was lost to follow-up after completing the needs assessment interview, so the study team enrolled an additional participant who completed the usability testing interview, 4-week trial period, and final interview to replace the individual lost to follow-up. See Table 1 for study participant details.

**Table 1:** Participant demographics

Participant #	Age (years)	Race and Ethnicity	Gender Identity	Diagnosis	LGBQ+ Status	Household Income	Insurance Status	At Risk for Food Insecurity
1	21	White, Non-Latinx	Female	Clinical/Subclinical Atypical Anorexia, Binge/Purge	LGBQ+	Less than $12,880	Public	Yes
4	52	White, Non-Latinx	Female	Clinical/Subclinical Atypical Anorexia, Binge/Purge	LGBQ+	$21,961-$26,500	Public	Yes
5	34	White, Non-Latinx	Female	Clinical/Subclinical Atypical Anorexia, Binge/Purge	Heterosexual	$21,961-$26,500	Public	No
6	36	White, Non-Latinx	Female	Clinical/Subclinical Atypical Anorexia, Binge/Purge	Heterosexual	$26,501-$31,040	Public	Yes
7	22	Black or African American, Non-Latinx	Non-binary	Clinical/Subclinical Atypical Anorexia, Binge/Purge	LGBQ+	$12,881-$17,420	Public	Yes
8	29	White, Non-Latinx	Male	Clinical/Subclinical Atypical Anorexia, Binge/Purge	LGBQ+	Less than $12,880	Public	Yes
9	29	Black or African American, Non-Latinx	Female/Non-binary	Subclinical Bulimia Nervosa	LGBQ+	$35,581-$40,120	Public	Yes
11	24	White, Non-Latinx	Female	Subclinical Bulimia Nervosa	Heterosexual	$35,581-$40,120	Public	Yes
12	70	White, Non-Latinx	Female	Unspecified feeding or eating disorder	Heterosexual	$65,001-$100,000	Public	No
13	68	White, Non-Latinx	Female	Binge Eating Disorder	Heterosexual	$65,001-$100,000	Public	No
14	38	American Indian or Alaska Native, Latinx	Female	Subclinical Bulimia Nervosa	Heterosexual	Less than $12,880	Uninsured	Yes

### Needs assessment

Feedback converged on three main themes. See additional examples of feedback displayed in Supplementary Table 1.

#### Recovery journey

##### Consequences of the ED

Participants exhibited self-awareness of their ED, highlighting consequences of their EDs across multiple areas of their lives, including social, physical, occupational, and emotional domains. One participant shared, “I didn’t have any friendships. I realized I didn’t have any skills to live on my own—how to pay bills, how to deal when things went wrong—just daily tasks I couldn’t cope with those without wanting to fall back on behaviors. I didn’t have any plans for my future,” (P8) voicing the serious toll his ED took on everyday functioning. Another participant explained, “[My ED] has impacted my life. It’s impacted my relationships and how I view myself, and probably my professional life as well” (P6).

##### Goals for recovery

Participants reported high motivation to pursue recovery in order to feel better about themselves and improve their quality of life. Goals often included remediating the consequences of their EDs, stopping ED behaviors, and developing new coping mechanisms. One participant noted, “I’d like to completely stop the binge-purge cycle and just be able to eat more throughout the day” (P5). Another participant commented on the importance of being able to effectively use coping skills in the face of eating disorder urges: “I feel like [developing] skills would probably be my most important goal right now…like these are the skills that I can use when I have the urge to restrict” (P1).

#### Treatment experiences

##### Barriers to treatment initiation and uptake

Participants reported numerous barriers to ED treatment uptake, including lack of insurance coverage, lack of geographic accessibility, and discrimination, which often intersected. One participant shared, “[Higher levels of care are] just not attainable—like, we have looked at every option—because of the type of insurance [Medicaid/Medicare] I have.” (P1). Another participant expressed, “As far as…finding…an outpatient or an inpatient program, that was difficult…. A lot of places—because I was overweight or my BMI was higher than a certain amount, even though I met other criteria—were like, ‘I don’t think this is the right fit’” (P4).

##### Poor quality ED treatment

Even when participants were able to receive ED treatment, many lamented poor quality of treatment, largely due to a lack of trained providers. One participant shared, “My current therapist is…not specifically an eating disorder therapist…[Good eating disorder therapists] are few and far between” (P7). Similarly, another participant noted, “With the therapist I see now…, I just feel like she doesn’t get it…. There’s definitely a difference between someone with the special licenses [to treat EDs] and someone who doesn’t have it” (P4).

##### General treatment experiences

Finally, participants shared experiences with treatment for other ED-related physical and mental health diagnoses. Participants discussed the difficulty of managing physical illnesses, such as diabetes and chronic liver injury, in the context of an ED. One participant underwent bariatric surgery, describing subsequently elevated ED cognitions in the aftermath that ultimately led to an ED relapse; others were contemplating bariatric surgery in the future. Additionally, participants reported co-occurring anxiety and/or depression, which were often the focus of treatment and precluded the ED from being addressed.

#### Experiences with and expectations for online programs

##### Past and present experiences with technology

Participants endorsed using multiple health-related apps, including My Fitness Pal, My Strength, Recovery Record, Weight Watchers, and Finch, as well as health technology, such as Fitbits and Apple watches. Participants had mixed feelings about whether technology had supported or impeded their ED recovery. For example, a participant shared, “I used to use My Fitness Pal. Not when I was like healthy dieting, or when it spiraled, but when I was trying to get…back on track for eating. I was trying to be a little more mindful of just…tracking it, but then that kind of backfired because when I saw it written out of like, ‘Oh, you ate this!’ Then it kinda just became detrimental instead of helpful” (P5), highlighting potential harms from app use despite recovery-oriented intentions.

Participants also shared aspects of the technology they did and did not like. One participant shared of Recovery Record (i.e., an app used to track meals alongside therapeutic support), “I think if I could take the time to do all of that, then it’s helpful. But sometimes…it just takes too long to answer all the little questions” (P4), expressing dislike for the number of prompts. On the other hand, the same participant noted that “It’s definitely better if you can connect it with someone who can look at it. Sometimes I think it’s helpful just where they [treatment team] can just see it, but you don’t have to like actually talk about, you know, as it’s hard to admit” (P4).

##### Future wants and needs for online program

Participants also gave feedback on what they hoped to see in an online program designed to facilitate ED recovery. For example, one participant shared, “Reminders or messages to kind of keep it in focus [would help keep me on track]” (P9). Participants also frequently endorsed the value of accountability with a coach in the program or peers going through a similar struggle. Furthermore, participants provided content recommendations, including adding content around managing co-occurring mental health conditions that could trigger ED behaviors.

### Usability testing

Usability testing feedback converged on three main themes. See additional examples of feedback displayed in Supplementary Table 2.

#### Content development

##### Specificity

In general, participants were enthusiastic about the range of topics covered, emphasizing that much of the content resonated with their experiences and was consistent with what they hoped to see in an online program. One participant shared, “I really like the goals, the values. I really like how the questions [about your relationships] made you really think about…who are these people and how you want them in your life, and you know, how you associate with them” (P14). Another participant explained, “I like the ‘balanced self talk.’ I like the examples of the ‘critical self’ because I think we don’t really think about those things on the daily” (P13). Others highlighted the need to strengthen the connection between the skills presented and specific eating disorder-related challenges, stating, “…what I’m reading here, although it’s great information, I’m not reading anything about this and an eating disorder…what can I do to help myself with my eating disorder?” (P13).

##### Inclusivity

Participants also commented on how to make the content more inclusive to people of various social identities. Many participants focused on the importance of representing people in bigger bodies and not just “the stereotypical eating disorder—like super skinny girl that thinks she’s fat, but she’s not” (P5). Participants also mentioned the importance of making the program relevant to men and gender diverse individuals.

##### Usability

Finally, participants offered suggestions to increase the usability of the program, including changing phrasing, breaking up text into smaller chunks, reducing the number of questions, and having audio options for long text passages. Participants also gave feedback about specific program tools. Regarding the meal tracking tool, one participant noted, “My first thought is…having a setting to click what are supposed to be your meal goals, because right now for me personally, we haven’t got into snacks, and we’re working on it” (P1), offering a suggestion to customize the tool. Overall, participants felt that the content was personally relevant and addressed an array of topics important for recovery; however, it did not necessarily reflect their social identities nor had the specificity needed to make the program feel tailored to eating and body related concerns.

#### Participant experiences with mental health

##### ED behaviors

Participants’ own ED behaviors directly informed their suggestions for improvement and their overall feelings towards the program content. One participant suggested that “[The program should cover] options for destroying of a scale… just to reiterate the point that weighing yourself is not a good idea” (P5). Another participant liked how the program approached exercise: “So I really like…that this takes a really balanced view with exercise. I like that it gives a variety of options for exercise [and]…. it allows me to think about physical activity in any sort of way. Not just thinking about it in a traditional way of going to the gym, or whatever” (P8).

##### Treatment exposure

Participants also shared their familiarity with different psychological skills and treatment modalities. For example, one participant expressed previous exposure to CBT and noted, “I feel that [identifying thinking patterns (e.g., catastrophizing)] is really useful…. I was in therapy before, for a couple of years and we talked about these kind of mentalities, and they’re definitely things that I had to deal with… to undo” (P14). Another participant indicated familiarity with the concept of meal tracking, and some concern about whether meal tracking would be focused on nutrition details and calories, or on feelings around eating (P7). Other elements of the program resonated as novel, as demonstrated by P4’s remarks on addressing physical activity: “I think I have not used another app, or anything that actually addresses exercise…in this way that I’m aware of. It might say, did you exercise or what did you do, but there’s nothing like, ‘Let’s come up with a plan.’” In general, participants found information presented in the program important and relevant, whether familiar or not.

#### Real-world use

##### Previous app use

One participant noted a dearth of apps focused on alleviating ED symptoms, sharing, “There are a lot of mental health apps out there, but there are not a lot of apps that are specific for eating disorders and challenging negative body image but also other aspects of your life” (P1), underscoring the importance of an online program focused on addressing EDs and broader life challenges simultaneously. Another participant expressed hesitation to use the program based on previous experiences with Recovery Record, sharing, “I hated Recovery Record because there were so many questions” (P5).

##### Anticipated CALM-ED engagement

Overall, participants indicated enthusiasm about using the program in their daily lives, and all endorsed that they would at least trial the program. One participant shared, “I totally would [use this program]. I think it’s different from… what I’ve used in the past…. I really like having a plan, whether it’s for like meal, planning, or exercise, or whatever” (P4). Another participant explained *how* she would use the program, noting, “Yeah, I definitely would use [the audio component of the program] because I spend a lot of time in my car, so if it was something that when I’m in my car, I usually listen to like podcasts and things like that” (P11).

## Discussion

The development and usability testing of a guided self-help digital CBT-based intervention for underserved adults with binge-type EDs fills a critical gap in advancing services for more diverse populations. The user-centered approach involved participants in each phase of the development process to increase relevance and prospective engagement, yielding several important insights [30]. Many participants identified multiple difficulties accessing evidence-based care. Participants described feeling discouraged about the lack of trained ED providers and frustrated with having to explain their illness and educate their providers about EDs, aligning with past literature that has elucidated barriers to treatment faced by publicly and uninsured populations [13, 15]. Notably, about half of the participants held one or more marginalized identities—most commonly queer, living with a physical disability, and having a larger body—which has been shown to pose unique challenges to receiving appropriate care [16]. Geographical location further impacted participants’ ability to connect with treatment. Ultimately, there were many factors that contributed to negative experiences seeking and engaging in treatment, all of which bolstered participants’ desire for an evidence-based, ED specific digital intervention.

Participants’ diverse identities and life circumstances affirmed that this program must address a breadth of unique obstacles faced by this population. In alignment with these experiences, feedback naturally centered around maximizing diversity and inclusivity throughout the program. Specifically, participants stressed the importance of including people with larger bodies and different abilities in examples of skills and accompanying infographics. They also emphasized improving accessibility throughout the program design, encouraging the addition of audio recordings and using graphics to streamline text-heavy sections, which could be reflective of education level [30]. Participants were excited about the coaching component, which has previously been demonstrated to facilitate program engagement [28], and wanted to connect with other users who have endured similar challenges to alleviate feelings of isolation [26]. A desire to cultivate a sense of community within the program, even if users weren’t directly engaging with others (e.g., direct messaging), reinforces the importance of social support in promoting recovery.

Limitations of this study include difficulties reaching the target population due to the complexities of public health care systems and potential sample bias. Given the known low rates of treatment seeking and engagement within the target population, this sample may have had unusually high treatment engagement and additionally did not reflect racial and ethnic diversity [5, 11, 31]. Future work should explore other recruitment avenues that may better reach the full spectrum of individuals with EDs who are under- or uninsured, including closer partnership with relevant community organizations or food banks. Lastly, the user-centered design approach could not be responsive to initial feedback on aesthetics and delivery (e.g., customizable color schemes, a phone app delivery) due to financial limitations and use of the Qualtrics platform.

## Conclusions

To date, this is the first digital CBT-based intervention adapted for populations at risk of experiencing health inequities, using an original program that has already been shown to be effective in other populations. A user-centered design approach was used, which enhances probable future engagement, and well-established guidelines for qualitative analyses were utilized [32, 33]. Finally, the participant pool included diversity in terms of age, geographic location, and income, capturing the experiences and perspectives of those who are under-resourced and underrepresented.

Preliminary results from the present study mirror emerging literature that mobile technologies have the ability to increase accessibility of treatment for those who have been historically precluded from accessing evidence-based ED care [33]. Participant feedback highlighted several key areas of focus in order to adapt existing EBIs to be appropriate for and acceptable to underserved diverse populations. These findings inform future program development and refinement, which will soon be tested in an open trial with a larger sample size. Continued testing will examine the effectiveness of this coached self-help program, which has the potential to address critical barriers to ED treatment for a population that is most in need of tailored evidence-based care.

## Electronic supplementary material

Below is the link to the electronic supplementary material.

Supplementary Material 1

Supplementary Tables 1 & 2

## Data Availability

The full dataset generated in this study is not publicly available to protect participants' privacy given the highly sensitive nature of the data. However, the data are available from the corresponding authors upon reasonable request.

## Abbreviations

BMI: Body mass index
CALM-ED: Changing Attitudes, baLance, and Mindfulness for Eating Disorders
CBT: Cognitive-Behavioral Therapy
EBI(s): Evidence-based intervention(s)
ED(s): Eating disorder(s)
IRB: Institutional Review Board
SES: Socioeconomic status
