# Supplementary material for: Adaptations of an online cognitive-behavioral therapy intervention for binge type eating disorders in publicly-insured and uninsured adults: a pilot study
Source: BMC Public Health. 2025 Apr 7;25:1296. doi: 10.1186/s12889-025-22494-w (PMC11974178; doi:10.1186/s12889-025-22494-w)
Supplement: Supplementary file 1 — Supplementary Material 1 [file 12889_2025_22494_MOESM1_ESM.docx]

**Supplemental Information**

**Program Outline**

1. **Session 1: Eating Well**

The first session invites the participant to create program goals and reflect on eating behaviors while introducing regular eating skills.

1. **Session 2: Coping Well**

Coping well addresses how to identify triggers, their impacts, and employ coping skills.

1. **Session 3: Thinking well**

Thinking well explores how thoughts impact feelings and how to shift negative thinking patterns.

1. **Session 4: Mind and Body Wellness**

Mind and body wellness examines body image, movement, and self-care.

1. **Session 5: Media Wellness**

Media wellness explores the role of social media’s influence on body image and the “idealized body”.

1. **Session 6: Relationship Wellness**

Relationship wellness discusses how to use effective communication skills to create, maintain, or terminate interpersonal relationships.

1. **Session 7: Emotional Wellness**

Emotional wellness examines how to identify core values, set behavior related goals that align with values, and how to cultivate a positive mindset.

1. **Session 8: Future Wellness**

Future wellness provides instructions on developing a relapse prevention plan and how to set SMART goals to promote recovery.

**Needs Assessment** **Interview Guide**

Hi, thank you for joining today!  I’m going to briefly explain the purpose of the interview and go over a couple of items before jumping in.

We are conducting this study to inform the creation of an online program for eating disorders, specifically focused on adults who have public insurance or don’t have insurance. We are hoping that this program will be a helpful resource for people who may not have quick access the kind of mental health care they need.

We need feedback from people who would be using the app to understand what your needs are and what you might want from an online program. This is the first time we are getting feedback so both positive and negative feedback is really helpful.

We are going to be touching on sensitive and personal topics that may be triggering or make you uncomfortable. Please let me know if you would like to take a break or skip a question that you do not want to answer.

We will be recording these interviews so that we can go back and take more detailed notes about what you share with us today. Do you have any questions, thoughts, or concerns about participating in this study, or recording the interviews?

**Background Questions:**

1. I’d like to start by learning a little bit about what prompted you to complete the online

screener about disordered eating.

1. How did you hear about this research study (i.e., NEDA, listserv, clinician, social media,

etc.)?

**Eating Disorder Behaviors:**

In that brief screener you completed, you reported that sometimes you [e.g., RESTRICTION: engage in dieting or try to cut back on what you’re eating, BINGE: lose control over your eating and feel that you eat too much; VOMIT/LAX: make yourself vomit, use laxatives or diuretics, engage in exercise, or engage in fasting). I’m going to refer to all of these behaviors collectively as disordered eating behaviors. Now I’m going to ask you more specifically about these behaviors.

1. How does [dieting/binge eating/vomiting or using laxatives] impact you, either positively

or negatively? (continue down this line of questioning)

- You mentioned (negative comments about dieting/binging/vomiting). Are there any other negative impacts of [dieting/binge eating/vomiting or using laxatives] for you?
- How much are you bothered by [reference some of the negative consequences]?

1. Have you ever received any mental health treatment or counseling for any of these

problems?

| If yes:   - What kind of treatment did you get? (individual therapy, group, higher level of care). - Tell me how that experience was for you. - Did you get the kind of help you were looking for? What was helpful? What was not helpful? | If no:  Could you tell me about what kept you from getting mental health treatment or counseling? |
| --- | --- |

1. Have you tried anything else so far to help you with your [dieting/ binge eating/ vomiting or laxative use]? Like talking to a friend, loved one, or a spiritual leader, a self-help approach, or anything else?

| If yes:   - What have you tried? - What have you found helpful? What hasn’t been helpful? | If no:  Could you tell me more about what’s kept you from trying anything yet? |
| --- | --- |

1. Do you have any current goals around your weight?
   - If yes: Tell me about your goals.
2. Do you have any current goals around your [dieting/binge eating/vomiting or laxative use]?
   - If yes: Tell me about your goals.
3. Do you have any current goals around your body image, and how you’d like to feel about your body weight or shape?

- If yes: Tell me about your goals.

1. Which of these goals is most important to you at this time (if they have stated they have multiple goals)?

**Technology Use:**

Now, I’m curious to learn more about how technology fits into your life.

1. Do you use any apps, social media, websites, online communities, or any other online resources to talk about or seek support for mental health or your eating/body image concerns?

| If yes:   - Tell me a little bit more about your use of [technology referenced]. - What do you use [technology] for? - How frequently and when do you use it? - Does it help? Why or why not?   **Repeat each set of questions for each different type of technology.* | If no:   - Can you tell me more about why you haven’t? - Have you ever used any technologies to support your mental health or eating/body image concerns? |
| --- | --- |

1. Are there any ways you’d imagine wanting to use technologies, like an app, to support you in [insert goals: developing a healthier body image, cultivating more balanced eating habits, managing urges to binge/vomit/use laxatives]?
2. What do you think would be most important for you to have in a program that would help you with your [goals: e.g., eating]?
3. Is there anything else you would want the program to do?
4. What kind of content, or information, would you want the program to provide?
5. Would you want to use it alone or with a coach or to interact with others?
6. How would you like to interact with this technology (e.g., daily, weekly, through messaging, etc.)?
7. How long do you think you would want to spend at a time on each module of the program (e.g., 30 minutes, 1 hour, etc.)?
8. Some people have trouble staying motivated to complete online treatment. Can you think of any specific ideas that would help you stay on track with the program?

**Current Program Review:**

We are developing a coached online program, and I’d love to get your thoughts on what’s in there so far.

1. Review outline of the current program:
2. **Session 1: Eating Well**

The first session invites the participant to create program goals and reflect on eating behaviors while introducing regular eating skills.

1. **Session 2: Coping Well**

Coping well addresses how to identify triggers, their impacts, and employ coping skills.

1. **Session 3: Thinking well**

Thinking well explores how thoughts impact feelings and how to shift negative thinking patterns.

1. **Session 4: Mind and Body Wellness**

Mind and body wellness examines body image, movement, and self-care.

1. **Session 5: Media Wellness**

Media wellness explores the role of social media’s influence on body image and the “idealized body”.

1. **Session 6: Relationship Wellness**

Relationship wellness discusses how to use effective communication skills to create, maintain, or terminate interpersonal relationships.

1. **Session 7: Emotional Wellness**

Emotional wellness examines how to identify core values, set behavior related goals that align with values, and how to cultivate a positive mindset.

1. **Session 8: Future Wellness**

Future wellness provides instructions on developing a relapse prevention plan and how to set SMART goals to promote recovery.

1. Are there any topics have we missed?
2. What within those topics would you expect to see or want to see?

**Prospective CALM-ED Use:**

What are your thoughts about this program? Do you think this is something you might use? Why or why not?

1. If yes: What would motivate you to want to use this program?

- Are there features that would be really important to you?
- Is there any specific content that would be really important to you?
- What do you think would keep you engaged in the program over time?

1. If no: Tell me more.

- Are there features of the program, or content, which would make it more appealing for you to use?

**Conclude Interview:**

Is there anything else that we haven’t talked about that you would want to see in this program or that would make it even more tailored just for you and the issues you are experiencing?

Thank you so much for taking the time to speak with me today and to provide your feedback on this program!

**Usability Testing Interview Guide**

As a reminder, this study is to get feedback on an online program for eating and body image concerns. This program is designed for people with eating disorders, specifically focusing on uninsured and publicly insured adults. We are hoping that this program will be a helpful resource for people who may not have access to the kind of mental health care they need.  Throughout this interview, I will show you different aspects of the program and ask for both positive and negative feedback.

Like the last interview, we are going to be touching on sensitive and personal topics that may be triggering or make you uncomfortable. Please let me know if you would like to take a break or skip a question that you do not want to answer.

We will be recording these interviews so that we can go back and take more detailed notes about what you share with us today. Do you have any questions, thoughts, or concerns about participating in this study, or recording the interviews?

To start, I’ll refresh you with a brief overview of the program modules. Each module will include:

1. A mindfulness exercise

2. An introduction of the topic

3. Educational content with infographics

4. An exercise to practice the skills

5. Reflection & critical thinking questions

Also, you’ll be able to communicate with your coach via text message, and only the coaching team will be able to see what you write in your responses on the program.

First, let’s review an outline of the sessions:

1. **Session 1: Eating Well**

The first session invites the participant to create program goals and reflect on eating behaviors while introducing regular eating skills.

1. **Session 2: Coping Well**

Coping well addresses how to identify triggers, their impacts, and employ coping skills.

1. **Session 3: Thinking well**

Thinking well explores how thoughts impact feelings and how to shift negative thinking patterns.

1. **Session 4: Mind and Body Wellness**

Mind and body wellness examines body image, movement, and self-care.

1. **Session 5: Media Wellness**

Media wellness explores the role of social media’s influence on body image and the “idealized body”.

1. **Session 6: Relationship Wellness**

Relationship wellness discusses how to use effective communication skills to create, maintain, or terminate interpersonal relationships.

1. **Session 7: Emotional Wellness**

Emotional wellness examines how to identify core values, set behavior related goals that align with values, and how to cultivate a positive mindset.

1. **Session 8: Future Wellness**

Future wellness provides instructions on developing a relapse prevention plan and how to set SMART goals to promote recovery.

**Questions:**

1. Was there anything you particularly liked?
2. Is there anything you wish we included?
3. Is there anything you particularly disliked?
4. Do you think this program could help others with similar struggles?

**Switch Screens to Program Screenshots:**

1. Ask initial thoughts after each screenshot; specifically:
2. Look and feel
3. Organization and navigation
4. Content

**Needs Assessment Interview Codebook**

**Likes**

**Dislikes**

**Suggestions**

**Willingness to use program**

**Goals**

- Body image
- ED behavior reduction
- Physical activity
- Weight loss
- Weight maintenance

**Barriers to care**

- Accessibility/insurance
- Discrimination
- Location
- Misdiagnosis of ED
- Unequipped providers

**Current technology use**

- Apps
- Miscellaneous online forums, websites, etc.
- Social media

**Experience with ED**

- Consequences of ED
  - Mental/emotional
  - Occupational
  - Physical
  - Social
- ED support
  - Current ED treatment
  - First time ED treatment
  - Prior ED treatment
  - Treatment modalities
- Years struggling/onset of ED

**Needs/wants for online program**

- Accessibility
- Aesthetics
- Coaching
- Content
- Ease of use
- Engagement
- Interactive activities
  - Autonomy/personal choice
- Social/community

**Non-ED diagnoses**

- Mental health
- Physical health
  - Bariatric surgery

**Reason for joining study**

- Contribution
- Tipping point (desperation for treatment)

**Usability Testing Codebook**

**Likes**

**Dislikes**

**Suggestions**

**Future program use**

- Accessibility
- Goals for using program
- Willingness to use

**Content**

- Inclusivity of different identities
  - Fatness
  - Culture
  - Disability
  - Body size
  - Gender identity
  - Sexual orientation
- Content diversity
- Applicable/resonant to participant needs
- Psych skills
  - Novelty
  - Previous exposure/use
- Specific examples
- Weight stigma

**Participant past/current experience**

- ED behaviors
- Other app use

**Program organization/structure**

- Ease of use/accessibility
  - Audio
- Coaching
- Aesthetics
- Interactive elements
  - Number of questions
  - Hyperlinks
  - Drop-downs
  - Questions/prompts
- Text
  - Length
  - Phrasing
