# Supplementary material for: Adaptations of an online cognitive-behavioral therapy intervention for binge type eating disorders in publicly-insured and uninsured adults: a pilot study
Source: BMC Public Health. 2025 Apr 7;25:1296. doi: 10.1186/s12889-025-22494-w (PMC11974178; doi:10.1186/s12889-025-22494-w)
Supplement: Supplementary file 2 — Supplementary Tables 1 & 2 [file 12889_2025_22494_MOESM2_ESM.docx]

**Supplementary Table 1.**

Themes, Descriptions, and Feedback from Needs Assessment Interviews.

| **Themes** | **Description** | **Illustrative Quotes (Participant #)** |
| --- | --- | --- |
| **Recovery Journey** | | |
| Consequences of ED | Participant input on the effects of their ED on their life. | “It feels like ED thoughts and my body weight...are kinda running my life. Like, it’s the most important thing.” (P7)  “I used to be able to like, remember things, and be on top of things. And it’s like now I just find myself struggling.” (P5)  “I’m more tired than normal, but I think there’s been a couple of days where I’ve really just...not been able to...go to work...and I think that was the big thing over the last couple of weeks.” (P4)  “I don’t participate in luncheons or eat when we have gatherings, and everybody is like, ‘Why do you not eat?’ I just say that I’m not hungry, and they kind of leave it alone. Everybody else is eating, but I’m just sitting and not participating.” (P6)  “I know now that I’m not as active as I used to be. I do ride horses twice a week, I try to swim twice a week, I mean it’s – I try. But my breath is not as good, and I’ve had it checked, so I know it’s my weight. I know my blood pressure is high because of my weight, so it certainly is impacting my health and it’s impacting me emotionally, too, because I know what I look like now and I don’t really want other people to see how I look. I’m embarrassed.” (P13)  “I just feel like I’m kind of spiraling right now with the stress levels and weight wise, and I just want to feel better. Right now, I feel bad all the time and I can feel myself gain five pounds. It just takes a big toll on me… I feel sick all the time. I am actually chronically ill, but just physically, I feel sick all the time, and I don’t like that.” (P9) |
| Goals for Recovery | Participants’ goals while pursuing ED recovery. | “I just wanna look in the mirror and think that I’m hot again. That’s my goal. I don’t really care what everybody else thinks…. I just want to feel better.” (P9)  “I want to be at least body neutral, that’s really my biggest goal. I just don’t want to feel anything about my body in general. It doesn’t have to be positive.” (P7)  “I would just like to get to a place where I don’t feel like saying no to a donut is the end of the world, like just feeling – I would love to just feel satisfied. That would just change my life. I don’t want to be thinking about food 24/7. That would be amazing.” (P11)  “It probably sounds weird, but I can’t even like run a mile. So it was like a goal of mine, for, a long time I would like to at least you know, participate in a 5k or something…I don’t think I’m gonna do a marathon, but you know…like, if I was to go on a trip or something, you know, like, I want to be able to do those things.” (P4)  “I just wanna be more in tune with any intuitive eating and providing for my body, and especially now it’s really important, because my daughter is 9, and the other day she was counting out her food like sorting like this and doing that. I’m like ‘Oh no.’… I don't want her going down the path.” (P5) |
|  |  |  |
| **Treatment Experiences** | | |
| Barriers to Treatment Initiation and Uptake | What got in the way of participants seeking and receiving ED treatment. | “I’m not even eating enough calories to sustain my body every day, but I’m overweight, and that’s all that people see. And so, they don’t realize that I’m malnourished or sick because they just see me as an overweight person.” (P9)  “Because I live in the middle of nowhere, it’s hard for me to get [to in-person support groups].” (P7)  “I’ve looked up a couple of things—like Googled, ‘Do I have an eating disorder?’ and take a little assessment and then it’s like, yeah, you probably do. Here’s some sites. And then I looked up one thing that came up on Google – the Emily project – but they aren’t local to me, and they’re like, ‘Oh, yeah, it’s like $9,000 a night.’” (P11)  “I mean care, even though it’s so amazing in the Bay Area, and we have Stanford and UCSF here, but we never have gone through insurance, and we’ve always been fortunate to have well trained people that have been very generous and have sliding scale spots. That’s the only way.” (P08)  “I’ve been trying to look into setting up a care team, but a lot of times I go through the insurance—the Medicaid website. And it’s like, ‘Oh yeah, these specialties,’ and you get the list, and then you start making the phone calls, and they’re like, ‘No, we don’t have a doctor that accepts that insurance. And we don’t do that, and we don’t have a location that does.’” (P5)  “I grew up – my parents are very religious, and they don’t believe in [mental health treatment]. They don’t really – they think that you know, taking medicine for your mental health is basically evil. So [seeking treatment] was not really an acceptable thing for me to do.” (P11)  “Somebody had told me about that, you basically get every doctor as if you were in like the treatment center… But, oh the prices on that were, whoa! As soon as…he was telling me about the two different programs and the amount you had to contribute, as far as how many days a week, and then the cost per day, it was like, ‘Yeah, no. Never in this lifetime.’” (P5)  “I just kind of made one big post about like, ‘This is what is going on with the way I eat. What in the world do I do?’. Pretty much everybody on Reddit was like, ‘yeah, you need to see a specialist. ‘You need to see a…professional’ and I was like, that's great. How? I don’t – where do I go? How many miles do I have to drive?” (P11)  “Oh, my gosh! [Finding new providers] has been so hard! I spend months and months and months… vetting the training that I’m looking for, and it’s taken years and years and years for me to try and find the resources to look for someone. And then when a therapist retires, or when it gets to a point for some reason why I need to switch, it gets really scary.” (P8) |
| Poor Quality ED Treatment | Negative experiences with ED treatment. | “Even now, I’ve been doing therapy for about four years now, and I don’t feel like it’s exactly what I need, but I also am aware of like the legal ramifications. I’m on Medicaid, so they don’t provide me the same services that they do for people that are paying out of pocket for these services...and I can feel that when I’m in therapy, they’re just listening and not really giving me the resources and that help that I need...so it’s been frustrating.” (P9)  “[My nutritionist] was rude from the start. I just felt like she saw me come in, looked over my body, and made up her mind as soon as she saw me. She took my blood pressure and then she started talking about how I probably need to adjust my diet. She told me that it was possible that in the stress that I was experiencing, that I forgot about my eating disorder because I gained so much weight.” (P9)  “I haven’t seen a lot of HAES [Health At Every Size]-informed providers. They’re all, ‘Lose weight or else you’ll die,’ and then I’m like, ‘This is my body. I’m here. I’m not dying.’” (P7)  “I brought my [eating] concerns up to my regular family physician, and she pretty much kind of said that there was no concern, and that she didn’t think I needed to get any referrals [to weight loss programs].” (P6)  “I already feel on a daily basis like I’m talking in another language to so many providers about this stuff, and I don’t wanna bring it up because I feel like I’m the crazy one. I just came from a new primary care provider, because I can’t find any good primary care providers now, unless I want to pay concierge because I’ve aged out of like the adolescent medicine clinics, and so I’m trying to talk to the primary care providers about trying to do the right labs, and they’re like ‘I don’t really think you need that.’” (P8)  “I feel like I’m a really complex case and just because someone knows about eating disorders doesn’t mean they are able to address all of the things. So, I’ve actually and more success with non-eating disorder clinicians. Actually, I’ve had a couple good eating disorder therapists, but they’re few and far between.” (P7)  “We focused on other things, cause I was a teen mom. I had my son when I was 13, and so I was pretty much kind of forced to go to [a therapist] by the state. That’s why I said that I think she’s kind of a multi range therapist and not say, an eating disorder specialist. Yeah, cause I don’t ever remember being classified as having a specific eating disorder. The only doctor seeing me… was for anemic and iron deficiencies.” (P6)  “My doctor – I talked to her about exercising. She said, ‘That’s not how you’re gonna lose weight.’ She said, ‘That’s going to make your heart strong but that’s not going to necessarily help you lose weight; it's your diet.’ And I said ‘Hmm!’ That was an odd thing to tell me.’” (P13) |
| General Treatment Experiences | How participants experienced treatment for various mental and physical health diagnoses. | “Well, I’ve had two different types of breast cancer. But, the second time, I had a double mastectomy, and I’ve not been able to have reconstructive surgery...they said it is too risky...unless my BMI is like 30 or below again, so I feel like that’s, you know, playing into [my eating disorder], like I still have all the excess fat from when I was 300 pounds.” (P4)  “So, I think that’s one thing I felt that was really good about Johns Hopkins is that I wasn’t the only one there that had had gastric bypass. You know at the time there was...at least 4 or 5 other people...So there it was they were saying that it's something that’s kind of common there.” (P4)  “And especially now because I’m diabetic, I really have to do better and like really take care of myself. It’s not really an option anymore, and I think what this is really about is if I don’t start doing better for myself, it’s just gonna be bad…I almost died in January. I was in DKA, which is diabetic ketoacidosis. By the time I got to the hospital, I was on the verge of going into cardiac arrest.” (P9)  “The psychiatrist did not seem to like my needs, or so I was told. I told him certain sleeping medications that just are not helpful for me. They cause really bad side effects, and he put me on the on one of the medications anyways…. A couple of antidepressants make me actively suicidal, and that’s what happened with this medication. So, I became actively suicidal with medication, and acted on, you know, a plan, and although I had kept telling them, ‘This is what’s going to happen,’ they put me on the medication.” (P7) |
| **Experiences with and Expectations for Online Programs** | | |
| Past and Present Experiences with Technology | How participants have used technology to improve their well-being. | “I use My Strength for my mental health...It’s an online app that’s... pretty much kind of article-based and activity-based, like it gives you multiple choice questions that you choose from, and then it gives you different topics and different articles and different suggestions and different helpful things.” (P6)  “I had a Fitbit watch, but there was an actual Fitbit—that app that kept track of all your calories, and so, with your steps in it, calculated your overall burned calorie rate compared to your intake, and all of that.” (P6)  “I have kind of been neglecting it recently, but before I [used the Finch app] every day, for...15 to 30 min at least. There are so many like little things you can do. There’s something called the Tree Town, where you can add people, and then you all like, get to see how you’re all doing as far as your self-care journey. I'm pretty sure there are other goals you can do. Also with Finch, they have Instagram, TikTok, a Discord server just for Finch, a Facebook group.” (P7) |
| Future Wants and Needs for Online Program | What participants hope to have in an online program focused on alleviating ED symptoms. | “I wonder if you could do something to set a reminder to make it be linked to your favorite song, or something, or have a positive connotation to customize it in ways that make it your own. If you could do it that way, or you could maybe upload a picture of how you view recovery. When people go to treatment, they bring pictures of loved ones and stuff like that, so I don’t know. That’s something that comes to mind.” (P8)  “The one thing I want to mention is having accessibility options in the app would be helpful like being able to have black, like dark screen, and being able to have the words speak if it’s like a very long question, being able to have speak the text, all of that that. Would be actually be helpful. And being able to like enlarge the text.” (P1)  “[Something that would keep me motivated is knowing] that someone else is gonna know if I’m not keeping up… even if it’s not my therapist, even if it’s someone else in that, that’s using the app that’s also doing it.” (P11)  “Being held accountable is one of my big influencers.” (P6)  “Every time we complete a session we get like a sticker. And we have to collect all the stickers to make the puzzle, or picture, or whatever like that. I love rewards.” (P1) |

**Supplementary Table 2.**

Themes, Descriptions, and Feedback from Usability Testing Interviews.

| **Themes** | **Description** | **Illustrative Quotes (P#)** |
| --- | --- | --- |
| **Content Development** | | |
| Specificity | Participants gave feedback about topics that were covered, as well as offered suggestions for other content. | “I like the behavioral strategies at the end, for a lot of people who are on social media, you know, especially the younger people. You know, ‘Set a timer, unfollow people’ because it can just become just its own spiral, so I think those are also useful.” (P8)  “That is so interesting that there can be such fluctuation in your weight every day. That’s really important [to know].” (P13)  “There are points in here where you say, ‘Exercise isn’t really about weight loss. Movement is a self-care routine, it’s about stress, dealing with chronic stress.’ I really like that.” (P8)  “So, it would help me if when you get down to catastrophizing and those things – how about adding some balanced self-talk there with those examples?... Because it starts off very minimal, well for me anyway, and then it just keeps building…so maybe, what could somebody say to nip that thought in the bud?” (P13)  “I think that the meal planning part is a little stressful for me. Planning out when and what you’re gonna eat, for me, it just feels like a lot of pressure. I don’t really eat at specific times every day, and I think planning out eating beforehand is hard for me, because I don’t usually know what I want to eat, or usually I’ll track my blood sugar before I eat and determine from there what I’m gonna do.” (P9)  “I think definitely putting culture in that pie chart would also be very important, like have a part with culture and the media and how they influence how people think about themselves.” (P7)  “I think we can expand a little bit on what values could mean… If I was to read that I didn’t really know what values were, I wouldn’t know what the hell you’re talking about.” (P14) |
| Inclusivity | Participants offered suggestions for making the content more inclusive. | “I don’t know how I feel about Jameela [Jamil]...It feels very weird, cause she’s in a thin body and… she says, ‘Oh, well, it’s not that important.’ But… she’s in a thin body. Like, it doesn’t impact her in ways that it does for maybe fat people.” (P7)  “Add a wheelchair [to the exercise section]. ‘Just do some laps on your wheelchair.’ Cause I use a wheelchair, so make it more inclusive movement.” (P1)  “I can understand from the viewpoint of someone that has body dysmorphia that is afraid of everyone thinking that they’re fat, but for people that are [fat]...I don’t think this is the most helpful example.” (P9)  “I feel like the ‘Exercises at Home’ section is kind of funky. It feels like that’s something [exercising at home] that not everyone can do or can’t do any of these exercises, because some people’s bodies just can’t.” (P7)  “Maybe have examples from…another gender.” (P11) |
| Usability | Participants commented on the various activities in the program, as well as phrasing of content, and provided recommendations to facilitate ease of use. | “Instead of giving people these options or things that most people do, maybe asking somebody, ‘What are your physical limitations,’ or like, ‘What are ways of moving your body in a way that feels good?’” (P7)  “Yes, [I would use the meal planning tool]. In the past, there have been times where I was really good about planning it, and then I might not always follow through, but if I have a plan, I am more likely to follow through. I have done something similar to this before where I would do a week at a time.” (P4)  “Having the option to [read and listen to content] is actually really helpful for me. I personally like to an option to listen and read at the same time.” (P7)  “I like [the audio clips], and especially for people that are dyslexic or just don’t feel like reading, I feel like that would be helpful to have audio clips.” (P9)  “How about giving me a link to an example of a video on YouTube that I can click and look at, you know, that I can see yeah, this is really fun.” (P13) |
| **Participant Experiences with Mental Health** | | |
| ED Behaviors | Participants discussed their experiences with their ED. | “I really like...that this [program] takes a really balanced view with exercise. I like that it gives a variety of options for exercise, and I struggle with doing physical activity because I always have relied on bingeing, purging, and restricting for weight loss, and I always hated physical activity, and so the more I think about it, it's like, ‘I just don't know what to do.’ What I like about this is that it allows me to think about physical activity in any sort of way. Not just thinking about it in a traditional way of going to the gym, or whatever.” (P8)  “Lately one of my biggest benefits...has been noticing the parts of me: my eating disorder part, and my other part that is me.” (P1)  “I don’t find myself squeezing a part of my body, but I do fixate on parts of my body I don’t like.” (P13) |
| Treatment Exposure | Participants shared what knowledge, skills, and therapeutic modalities they had been exposed to in treatment. | “I guess this is beneficial to people that don’t realize that information. I mean I already knew it.” (P5) |
| **Real-World Use** | | |
| Previous App Use | Participants talked about how they previously used apps. | “With...Recovery Record...[meal tracking] is focused on the feelings.” (P7)  “I think I told you about Recovery Record and how much I hated that because of how many questions.” (P5)  “One thing that was helpful for me in Recovery Record was that sometimes I wouldn’t bring up certain things, but I would write it in the app, and then [my therapist] could still see it. It took away the courage of having to say something aloud.” (P4)  “I feel like it [Recovery Record] is helpful to track urges, like it would ask if you were having urges, and then you would have to rate it like a certain level, so if you’re taking laxatives, it would ask you how many, since your last log, which is important.” (P4)  “[Noom] had you weigh yourself every day. And that was, I think that probably isn’t the best behavior for me.” (P11) |
| Anticipated CALM-ED Engagement | Participants shared how they anticipated using the CALM-ED program in their everyday lives. | “I think it’ll take getting in the habit of it, but I do [see myself using the app]. [The structure] is nice.” (P8)  “I don’t know about the longevity of using [the program]. Like, I would use it try it out, but I don’t know.” (P5)  “I totally would [use this program]. I think it’s different from kind of what I’ve used. At least what I’ve used in the past...I really like having a plan, whether it’s for like meal, planning, or exercise, or whatever. I really like that.” (P4)  “My life doesn’t seem very regular, so [meal] planning out more than probably three days in advance is all I would be able to stick to, I think. Just because of the circumstances.” (P11)  “Yeah, I definitely would use [the audio component of the program] because I spend a lot of time in my car. When I’m in my car, I usually listen to like podcasts and things like that.” (P4) |
